# Streamlining statistical reproducibility: NHLBI ORCHID clinical trial results reproduction

**DOI:** 10.1093/jamiaopen/ooac001

**Published:** 2022-01-14

**Authors:** Arnaud Serret-Larmande, Jonathan R Kaltman, Paul Avillach

**Affiliations:** 1 Department of Biomedical Informatics, Harvard Medical School, Boston, Massachusetts, USA; 2 Computational Health Informatics Program, Boston Children's Hospital, Boston, Massachusetts, USA; 3 Division of Cardiovascular Sciences, National Heart, Lung, and Blood Institute, NIH, Bethesda, Maryland, USA

**Keywords:** clinical trial, statistical reproducibility, FAIR principles

## Abstract

Reproducibility in medical research has been a long-standing issue. More recently, the COVID-19 pandemic has publicly underlined this fact as the retraction of several studies reached out to general media audiences. A significant number of these retractions occurred after in-depth scrutiny of the methodology and results by the scientific community. Consequently, these retractions have undermined confidence in the peer-review process, which is not considered sufficiently reliable to generate trust in the published results. This partly stems from opacity in published results, the practical implementation of the statistical analysis often remaining undisclosed. We present a workflow that uses a combination of informatics tools to foster statistical reproducibility: an open-source programming language, Jupyter Notebook, cloud-based data repository, and an application programming interface can streamline an analysis and help to kick-start new analyses. We illustrate this principle by (1) reproducing the results of the ORCHID clinical trial, which evaluated the efficacy of hydroxychloroquine in COVID-19 patients, and (2) expanding on the analyses conducted in the original trial by investigating the association of premedication with biological laboratory results. Such workflows will be encouraged for future publications from National Heart, Lung, and Blood Institute-funded studies.

## Lay Summary

The COVID-19 pandemic has seen several articles published in high-profile journals being retracted. These retractions undermined even more confidence in the peer-review process, which is not considered sufficiently reliable to generate trust in the published results. A significant number of these retractions occurred after in-depth scrutiny of the methodology and results by the scientific community. This partly stems from opacity in published results, the practical implementation of the statistical analysis often remaining undisclosed. This article presents a simple workflow that leverages a combination of preexisting and newly developed biomedical informatics tools to promote transparent statistical analysis in biomedical research, which relies on the National Heart, Lung, and Blood Institute (NHLBI) BioData Catalyst platform. By streamlining access to data and analysis source code, it eases results reproduction and accelerates supplemental analyses. Such workflows will be encouraged for future publications from NHLBI-funded studies. We illustrate it by reproducing the results of the ORCHID clinical trial, which evaluated the efficacy of hydroxychloroquine in COVID-19 patients.

As of February 7, 2021, the website retractionwatch.com referenced 69 retracted COVID-19-related papers. This recent spate has highlighted an increasing mistrust in biomedical research overall, both by scientists and general audiences. Peer review alone is insufficient to validate the reported results. This realization is heightened as some retractions occurred in the most prestigious medical journals. This mistrust is a further reflection of the ongoing reproducibility crisis in biomedical research. Therefore, as a proof of concept, we present how we leveraged a biomedical informatics platform (BioData Catalyst powered by PIC-SURE) we developed for the National Heart, Lung, and Blood Institute (NHLBI) to reproduce the results of a recently published clinical trial evaluating the efficacy of hydroxychloroquine on patients hospitalized for COVID-19.

The numerous publications on the reproducibility crisis have sounded an alarm, leading to the emergence of multiple guidelines to tackle the problem, including the use of large sample sizes, multiple-comparison accounting, preregistration of research hypotheses, and standardization of reporting guidelines.^1^^,^^2^^,^^3^

From a bioinformatics perspective, a strong emphasis has been placed on sharing data and statistical analysis source code to enable the research community to internally validate the results. Despite the encouragement of source code sharing, the most influential medical journals have not mandated the practice.^4^

A second enabler of reproducibility is making experimental data accessible. Sharing medical data is critical to scientific knowledge dissemination; however, it conflicts with individual privacy concerns.

Given these two constraints, others have proposed openly shared anonymized datasets and data sharing by request of investigators. However, these solutions are suboptimal; a better answer lies in dedicated health data repositories offering centralized, controlled-access to sensitive data. Examples of such repositories include the NCTN/NCORP Data Archive for datasets from clinical trials of the National Clinical Trials Network (NCTN), the NCI Community Oncology Research Program (NCORP), or the UK Data Service.^5^ However, the use of such solutions is not yet widespread because sharing data conflicts not only with individuals' privacy but also with investigators’ direct interests. Building a cohort involves a significant time and cost investment; thus, investigators are reluctant to share it. As a consequence, data sharing through standardized means remains relatively limited, despite substantial funding.

A recent study examined the proportion of clinical trials that share their data after publication. Of 487 clinical trials published in three of the most influential medical journals—*NEJM*, *Lancet*, and *JAMA*—only 17 (3.5%) shared data through repositories, even though 89 had pledged to do so during the publication process.^6^ Clearly, there is a desperate need for broader data sharing, amplified by the recent questioning of the internal validity of some COVID-19 studies.

In response to this concern, we present a workflow by which we reproduced the NHLBI-funded ORCHID clinical trial.^7^ This is a multicenter, randomized controlled clinical trial comparing hydroxychloroquine against placebo, which took place between April and July 2020 in the United States. The primary outcome was patients’ clinical status assessed at 14 days after inclusion.

The analysis reproduction process took advantage of NHLBI BioData Catalyst. This ecosystem has been created to ease data reuse of NHLBI and other NIH-funded studies, providing a set of tools for efficient data exploration, analysis, and reporting. Data are managed in a secure, cloud environment and can be explored using graphical user interfaces, or retrieved through an application programming interface (API), thereby enforcing high standards in cybersecurity and mediating user-specific data access authorization, in this case through the PIC-SURE API. The API is accessible via two different open-source programming languages clients, R and python. The NHLBI encourages investigators to contribute to the BioData Catalyst ecosystem by making their source code available at the time of publication, preferentially through an investigator-friendly format like a Jupyter Notebook. It is a format that combines plain text, source code, and outputs in a single file, widely used in the data science community and already described as a valuable tool to make analysis reproducible.^8^^,^^9^

**Figure 1.**
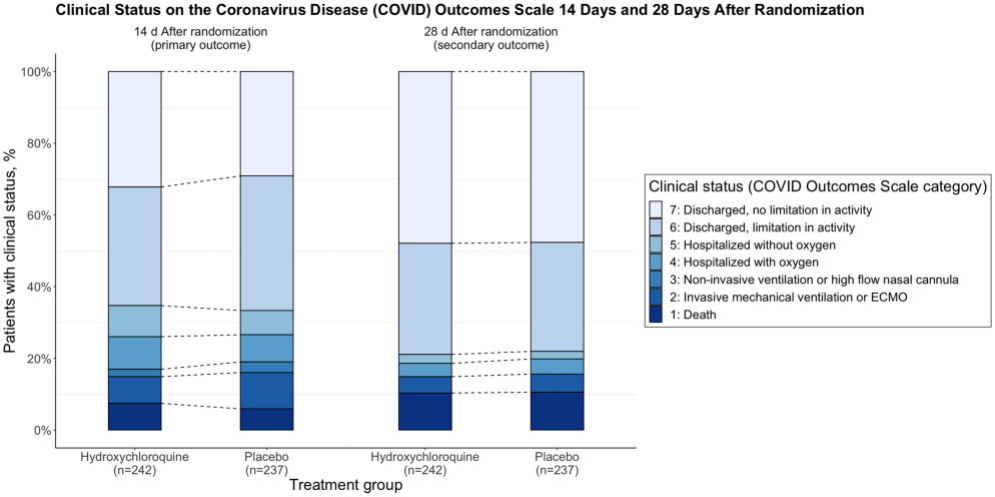
Primary outcome of the ORCHID Clinical Trial. The top panel represents the bar plot as published in the original article. The bottom panel is the figure as displayed in the notebook reproducing the analysis.

We accessed the data of the ORCHID clinical trial using the BioData Catalyst powered by PIC-SURE API and reproduced the entire analysis using the R programming language (R 4.0.3). This reproduction is based on the original investigators’ source code, consisting of several SAS analysis source files, and the statistical analysis plan. From data retrieval to plotted results, the entire process is displayed in the form of a Jupyter Notebook, freely available on GitHub.^10^ All the published results were identically reproduced (Figure 1), except for one safety outcome (lymphopenia was mistakenly reported instead of cytopenia). The authors acknowledged the error, noting that a typographical error caused it in the data management source code. Other than this mistake, our results and interpretation are identical to those published in the original article: hydroxychloroquine did not demonstrate superior performance to placebo in hospitalized patients with COVID-19. The reproduction of results of an RCT based on its published protocol, although seemingly straightforward, is far from granted: a systematic review found out that discrepancies or selective reporting are common between the published analysis of an RCT and what was initially planned in the protocol.^11^

**Figure 2.**
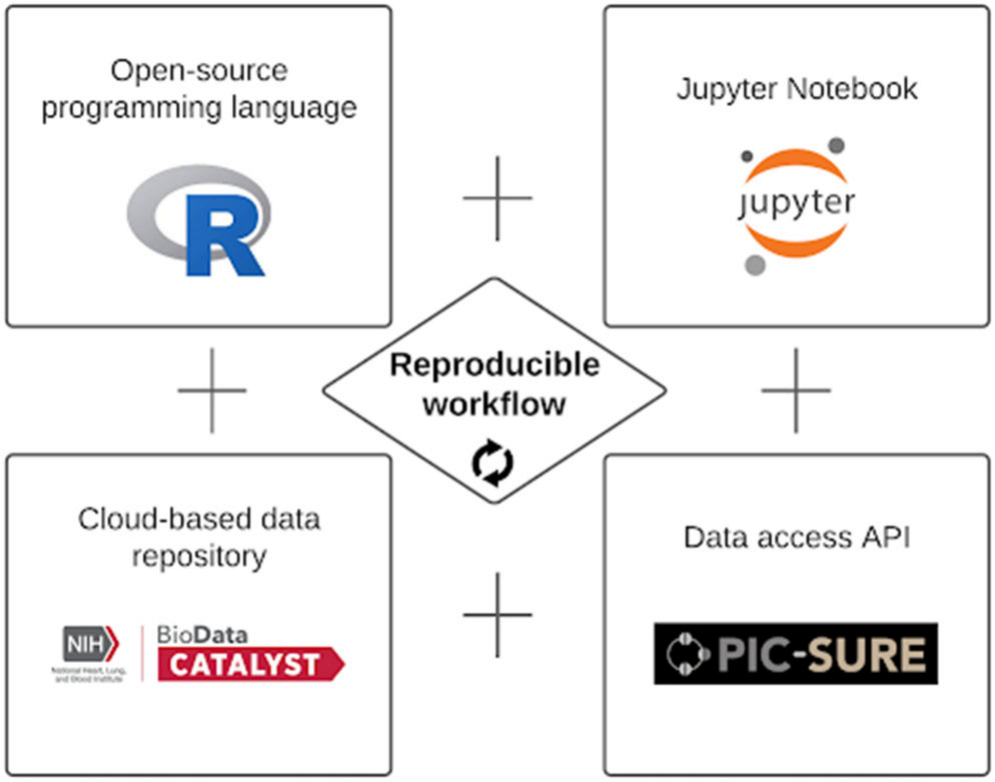
Elements composing the reproducible workflow.

Leveraging preexisting and newly developed tools, our work showcased how the combination of simple standard tools (open-source programming language, notebook, data repository, and API) can streamline the reproducibility process (Figure 2). Moreover, by using the cloud computing environments provided by BioData Catalyst, any investigator registered in the ecosystem and authorized to access this dataset in the database of Genotypes and Phenotypes (dbGaP) can execute the notebook in one click, without the need for downloading the data. Finally, because the heavy lifting data management process has already been done, it lowers the entry cost for investigators who can reuse the data and code right away and kick-start new analyses.

**Figure 3.**
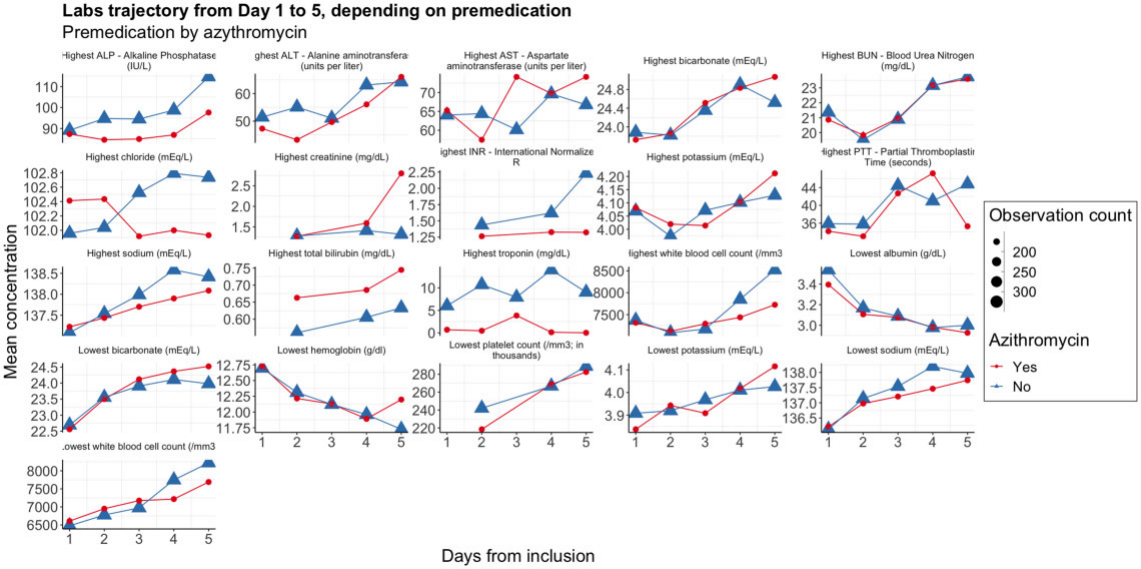
Supplemental analysis based on ORCHID clinical trial data: laboratory test trajectories according to premedication by azithromycin.

We illustrate this idea by carrying out sensitivity analyses that extend the work realized by the original investigators. We studied the correlation of premedication by azithromycin and outcomes (treatment received before entering the clinical trial). This off-label drug has been commonly used as a COVID-19 treatment, especially at the beginning of the pandemic (150 out of 479 patients of the ORCHID clinical trial population received it before inclusion).^12^ Our analysis showed that baseline clinical status as well as outcomes were worse in the population of patients who received azithromycin, possibly reflecting an incentive to use off-label drugs on more severe cases (Table 1).^13^ We also studied the differences in the lab trajectories during the first 5 days of the clinical trial, according to having received azithromycin. Systematic differences can be observed in AST, ALP, ALT, and troponin concentrations among the laboratory values studied (Figure 3). More extreme values are present in the group that did not receive azithromycin, highlighting the baseline characteristic differences in patient groups. One potential explanation could be the lower rate of prescription of azithromycin to patients with liver or cardiac conditions.^14^

**Table 1.** Sensitivity analysis: COVID-19 Outcomes Scale at randomization, day 14, and day 28, according to premedication by azithromycin (prescription of azithromycin before inclusion in the trial)

	At randomization		14 d after randomization		28 d after randomization	
COVID-19 Outcomes Scale	Yes (*n* = 150)	No (*n* = 329)	Yes (*n* = 150)	No (*n* = 329)	Yes (*n* = 150)	No (*n* = 329)
(1) Death	0 (0%)	0 (0%)	10 (6.7%)	22 (6.7%)	17 (11.3%)	33 (10%)
(2) Invasive mechanical ventilation or extracorporeal membrane oxygenation	16 (10.7%)	16 (4.9%)	20 (13.3%)	22 (6.7%)	12 (8%)	11 (3.3%)
(3) Noninvasive ventilation or high flow nasal cannula	22 (14.7%)	33 (10%)	3 (2%)	9 (2.7%)	0 (0%)	0 (0%)
(4) Hospitalized with oxygen	69 (46%)	155 (47.1%)	11 (7.3%)	29 (8.8%)	4 (2.7%)	15 (4.6%)
(5) Hospitalized without oxygen	43 (28.7%)	125 (38%)	8 (5.3%)	29 (8.8%)	3 (2%)	8 (2.4%)
(6) Discharged, limitation in activity	0 (0%)	0 (0%)	63 (42%)	106 (32.2%)	50 (33.3%)	97 (29.5%)
(7) Discharged, no limitation in activity	0 (0%)	0 (0%)	35 (23.3%)	112 (34%)	64 (42.7%)	165 (50.2%)

An essential part of producing sound and reproducible analyses lies in following established guidelines when reporting results. Our work implements the FAIR principle, materialized by the Three-point FAIRification Framework: findable (data loaded in BioData Catalyst is being assigned globally unique and persistent identifiers, and variable names are searchable globally without requiring specific authorization), accessible (the API and web-based graphical user interface are implemented in open source languages, with different level of authorizations), interoperable (data vocabulary and variables are being made accessible through open PIC-SURE), and reusable (reusable by every person granted individual patient level access). A more detailed version of these principles is provided in Table 2, and the FAIR initiative website provides a checklist for investigators who want to implement these principles.^15^

**Table 2.** Details of the “FAIR Guiding Principles for scientific data management and stewardship”

FAIR principles	Details
Findable	F1: (Meta)data are assigned a globally unique and persistent identifier F2: Data are described with rich metadata (defined by R1 below) F3: Metadata clearly and explicitly include the identifier of the data they describe F4: (Meta)data are registered or indexed in a searchable resource
Accessible	A1: (Meta)data are retrievable by their identifier using a standardized communications protocol A1.1: The protocol is open, free, and universally implementable A1.2: The protocol allows for an authentication and authorization procedure, where necessary A2: Metadata are accessible, even when the data are no longer available
Interoperable	I1: (Meta)data use a formal, accessible, shared, and broadly applicable language for knowledge representation I2: (Meta)data use vocabularies that follow FAIR principles I3: (Meta)data include qualified references to other (meta)data
Reusable	R1: (Meta)data are richly described with a plurality of accurate and relevant attributes R1.1: (Meta)data are released with a clear and accessible data usage license R1.2: (Meta)data are associated with detailed provenance R1.3: (Meta)data meet domain-relevant community standards

It is worth noting that the advancements presented in our work are primarily addressing the statistical aspect of reproducibility. An entire body of literature has been devoted to identifying and handling other elements of reproducibility. Setting up reproducible environments is an essential part of reproducibility, but it usually involves a certain degree of technicity. Almugbel et al^16^ have facilitated the use of containers by setting up a web-based interface automatically generating Dockerfiles. Eyal-Altman et al^17^ created a platform (PCM-SABRE) for reproducing and expanding on previous work in the domain of prediction in oncology. Reproducibility issues can also stem from experimental design, selective reporting, and journal publication biases. Study preregistration has been thought of as a potential solution for these issues.^18^

Nonetheless, the presented solution represents a significant step forward in reproducibility by providing NHLBI's investigators the possibility to reuse data and transparently showcase their work. But the impact of such a process actually comes from the level of adoption by the research community. As a comparison, publishing a clinical trial protocol on clinicaltrials.gov has become ubiquitous in the medical research community because it addressed a crucial need of the scientific community: selective reporting of results and data-driven inferences. Similarly, such an initiative will only be helpful if embraced by the research community on a large scale. Therefore, we advocate that the practice of submitting a report of the analysis code in the form of a Jupyter-like format could be encouraged by updating the clinical trial reporting guidelines.^19^ Moreover, medical journals could also make it a required document for publication, especially because the minimal requirements to implement such workflow only rely on a few elements: using an open-source programming language, displaying the code and results in a notebook, and accessing the data from a cloud-based repository. Such initiatives would go a long way in incentivizing investigators to produce robust analyses, thereby fostering trust in published results.

Besides BioData Catalyst, other initiatives emphasize reproducibility through transparent workflow and results, such as AnVIL, a genomic data resource that leverages a cloud-based infrastructure for democratizing genomic data access, or the Cancer Research Data Commons that connects diverse datasets with analytical tools in the cloud. Over the course of the COVID-19 pandemic, the NIH launched the National COVID Cohort Collaborative which aims at sharing and harmonizing individual-level clinical data to accelerate COVID-19 research, and in which transparency and reproducibility are promoted as cornerstones of the project and facilitated by cloud-based platforms and tools.^20^ Moreover, the NIH set up the Cloud Platform Interoperability Effort to allow investigators using BioData Catalyst data to find and integrate data across three other platforms: Cancer Research Data Commons, Kids First Data Resource Center, and AnVIL, therefore making BioData Catalyst part of a larger network of connected cloud-based data repositories.

We do not anticipate that our effort will solve the reproducibility crisis. The practices laid out here are only effective if combined with other best practices for conducting reproducible science. It is worth noting that this protocol cannot help with flaws intrinsic to a given study, like data collection issues, flaws in the study design, or population selection bias. Neither can it eliminate the necessary reproduction of experimental results using different populations or variations in the methodology. Although these issues are of paramount importance, they would be addressed by a completely different framework. However, the principles demonstrated can address reproducibility issues that stem from inaccuracies in statistical analysis or data-management process; discrepancies between prerecorded and shared outcomes; selective reporting; and other practices like p-hacking or outliers trimming.^1^

Those principles can have a real impact. The heated debate over hydroxychloroquine may have benefited from more transparency in the analyses, helping science prevail over opinion and eventually translating into more informed treatment choices and public health policies.

## Funding

This work was supported by the National Institutes of Health, National Heart, Lung, and Blood Institute, through the BioData Catalyst program (award 1OT3HL142479-01, 1OT3HL142478-01, 1OT3HL142481-01, and 1OT3HL142480-01).

## Author Contributions

PA and JK conceptualized the work; PA setup the software platform to access the data; ASL and PA designed the methodology; ASL carried out the statistical analyses; PA supervised the statistical analyses; ASL and PA accessed and verified the underlying data; ASL, JK, and PA interpreted and validated the results; ASL drafted the initial version of the manuscript; ASL, PA, and JK reviewed and edited the manuscript. All authors approved the final version to be published. All authors agreed to be accountable for all aspects of the work in ensuring that questions related to the accuracy or integrity of any part of the work are appropriately investigated and resolved.

## Ethics Approval

This study was deemed institutional review board (IRB) exempt by the Harvard Medical School IRB based on its being nonhuman subject research.

## Data Availability

Original data of the ORCHID clinical trial are available on Biodata Catalyst under the accession number phs002299.v1.p1. Information to get dbGaP access approvals is available at https://biodatacatalyst.nhlbi.nih.gov/covid-19.
